# Physician’ entrepreneurship explained: a case study of intra-organizational dynamics in Dutch hospitals and specialty clinics

**DOI:** 10.1186/1478-4491-12-28

**Published:** 2014-05-19

**Authors:** Wout T Koelewijn, Matthijs de Rover, Michel L Ehrenhard, Wim H van Harten

**Affiliations:** 1 Department of Health Technology and Services Research, School of Management and Governance, University of Twente, Drienerlolaan 5, Enschede 7522, NB, The Netherlands; 2 Netherlands Institute for Knowledge Intensive Entrepreneurship (NIKOS), School of Management and Governance, University of Twente, Drienerlolaan 5, Enschede 7522, NB, The Netherlands

**Keywords:** Hospitals, Specialty clinics, Physicians, Managers, Entrepreneurship

## Abstract

**Background:**

Challenges brought about by developments such as continuing market reforms and budget reductions have strained the relation between managers and physicians in hospitals. By applying neo-institutional theory, we research how intra-organizational dynamics between physicians and managers induce physicians to become entrepreneurs by starting a specialty clinic. In addition, we determine the nature of this change by analyzing the intra-organizational dynamics in both hospitals and clinics.

**Methods:**

For our research, we interviewed a total of fifteen physicians and eight managers in four hospitals and twelve physicians and seven managers in twelve specialty clinics.

**Results:**

We found evidence that in becoming entrepreneurs, physicians are influenced by intra-organizational dynamics, including power dependence, interest dissatisfaction, and value commitments, between physicians and managers as well as among physicians’ groups. The precise motivation for starting a new clinic can vary depending on the medical or business logic in which the entrepreneurs are embedded, but also the presence of an entrepreneurial nature or nurture. Finally we found that the entrepreneurial process of starting a specialty clinic is a process of sedimented change or hybridized professionalism in which elements of the business logic are added to the existing logic of medical professionalism, leading to a hybrid logic.

**Conclusions:**

These findings have implications for policy at both the national and hospital level. Shared ownership and aligned incentives may provide the additional cement in which the developing entrepreneurial values are ‘glued’ to the central medical logic.

## Background

In the Netherlands, a system of regulated competition with a mandate for individuals to purchase insurance was introduced in 2006. In addition, reforms containing elements from managed competition were implemented as the former lump-sum financing system was gradually replaced by a fee-for-service system. This, combined with the favourable treatment of specialty clinics, caused an increase in entrepreneurial activities. The number of specialty clinics (many of which were founded by physician-entrepreneurs) rose by 62% from 149 in 2007 to 241 in 2010, while the total revenue of these clinics tripled to 315 million euros, equaling roughly 2.5% of the total hospital budget [1]. These developments were supported by the Dutch Healthcare Authority, which advocated more specialty clinics because of their substantially lower charges compared to hospitals [1], while in addition, the Dutch Health Care Inspectorate pointed at the positive developments with regard to the quality of care provided by specialty clinics [2,3]. In the media as well, specialty clinics were being welcomed as efficient providers, providing fast access to patient-oriented care [4-7].

Despite the positive perception of physicians’ entrepreneurship as a part of managed competition, it still remained a relatively rare phenomenon as the vast majority of physicians continued to work in hospitals, leaving the start of specialty clinics to the ‘entrepreneurial few’. At times, the entrepreneurial ambitions of these physicians resulted in fierce conflicts with hospital management who felt surprised by their aspirations [8,9]. This triggered the questions answered in this paper: What drives physicians’ entrepreneurship? In answering this question we will adopt a neo-institutional perspective focusing on both contextual- as well as intra-organizational dynamics.

Over time, the rising demand for care, the continuous stream of technological innovations and health care reforms, reinforced by the financial crisis, have strained the relationship between physicians and managers in many hospitals [10,11]. This relationship, however, embodies ‘a critical determinant of the success of health care organizations’ [12-16].

On a more fundamental level, the relationship between physicians and managers is influenced by institutional logics [17], consisting of ‘taken-for-granted rules’; these are influential in shaping both organizational fields, such as health care, and the behaviour of organizations, groups, and individuals working in these fields [18]. In general, physicians are embedded in the traditional logic of medical professionalism, which includes values like external orientation and physicians’ autonomy. In contrast, hospital managers are embedded in the logic of business-like health care [19], focusing on values such as efficiency, and performance and quality indicators [17]. These different logics and accompanying value commitments are competing for dominance and lead to rivalry between the two groups. [16,17,19-22].

**Figure 1:**
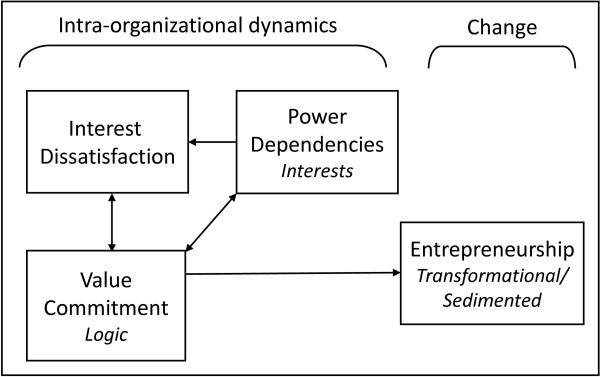
**Neo-institutional dynamics model based on Greenwood and Hinings**[15].

Neo-institutional theory allows for further analysis of these rivalries between groups within organizations. In particular, the framework by Greenwood and Hinings [23] is well-suited to study intra-organizational dynamics. Figure 1 depicts how intra-organizational dynamics, consisting of power dependencies, interest dissatisfaction, and value commitments, may lead to radical organizational change. Next, we briefly explain our core constructs and their meaning in a health care context.

The growing dominance of business-like health care at the expense of the traditional logic of medical professionalism has altered physicians’ perceived power dependence on management. It has decreased physicians’ autonomy, potentially leading to interest dissatisfaction - especially for physicians embedded in the logic of medical professionalism. Greenwood and Hinings [23] define such interest dissatisfaction as ‘the degree of dissatisfaction of groups and individuals with the existing distribution of resources and their motivation to enhance or sustain their shares of scarce and valued resources’. An increase in interest dissatisfaction will influence physicians’ value commitments.

Value commitments vary according to their embeddedness in a certain logic [23]. In the case of ‘*status-quo* commitment’ and ‘indifferent commitment’ there will only be limited if any intra-organizational dynamics, since organizational groups are respectively committed to the *status quo* or are indifferent about which logic is dominant. However, when all organizational groups agree a change of logic is necessary they have a ‘reformative commitment’. Alternatively, when one group challenges the dominance of the logic held by another group there are ‘competitive commitments’ and there will be strong intra-organizational dynamics.

A competitive value commitment may be either transformative or defensive in orientation [24]). In the hospital context, physicians holding a transformative oriented competitive value commitment favour the newly dominant business logic over the traditionally dominant medical logic. This contrasts with the opposite situation of physicians holding a defensively oriented competitive value commitment.

The interplay of power dependence, interest dissatisfaction, and value commitments can result in ‘radical change’, which involves abandoning the current dominant logic rather than fine-tuning it. In this study we define entrepreneurship as a form of radical change by physicians (also see, Koelewijn *et al*., 2012) [24]. Hereby, entrepreneurship is defined as ‘new entries by physicians and hospital managers who discover, evaluate, and exploit opportunities to create future health services by bearing the risk of profit and loss’ [25-27].

Finally, in analyzing the nature of radical change towards entrepreneurship, we distinguish between transformational and sedimented change [28]. Whereas transformational change entails the creation of an entirely new logic, sedimentation suggests that elements from another, sometimes conflicting, logic may be added to the present logic. As a result, this type of change remains incomplete and reversible [29]. Examples of sedimented change are provided by, for example, Kitchener [30], who applied Greenwood and Hinings’ (1996) model in explaining the introduction of a quasi-market in the UK. He concluded that this did not lead to a transformational change in the logics of its actors, but instead to a ‘co-existence of new structures and systems’. This was supported by Addicott and Furlie [31] who concluded that in managed clinical networks for cancer in London ‘a hybrid interpretive scheme has prevailed, whereby the characteristics of a range of conflicting archetypes coexist’. By analyzing the type of change associated with physicians’ entrepreneurship, we simultaneously contribute to the stream of literature on professionalism [32] in which professionalism is defined as a normative value system and ideology [33].

## Methods

In preparing our interview protocol, we made one adaptation to the original model of Greenwood and Hinings (1996) [23]. As both the dominant logic and the alternative logic are well defined in our study (business-like health care and medical professionalism respectively), we focus on both the physicians’ relative position with respect to the two logics and on defining their value commitment towards the other group. For this purpose, the description of the medical logic and business-like logic as developed by Reay and Hinings [8] was used. Respondents were assigned to one of the two logics based on their responses to the questions, which contained statements referring to values of both the medical logic and the business logic. Respondents who embraced values of both logics were assigned to the hybrid logic. The resulting research model is shown in Figure 1.

We conducted a study among physicians and managers in both hospitals and specialty clinics. We conducted semi-structured interviews with fifteen physicians and eight managers in four Dutch hospitals and twelve physicians and seven managers in twelve Dutch specialty clinics. In total, we interviewed 27 physicians and 15 managers to determine the intra-organizational dynamics, their contribution to the level of entrepreneurship, and if any resulting change was transformational or sedimented.

As indicated in Table 1, the four hospitals and twelve specialty clinics ranged in size and specialty, thereby constituting a representative sample of hospitals and specialty clinics in the Netherlands. Per hospital, we selected two physicians from medical specialties and two from surgical specialties. Managers were selected for having a direct relation with at least two of the physicians included to allow for a better understanding of the intra-organizational dynamics. For specialty clinics, we focused on including physicians from clinics of differences sized rather than from different specialties.

**Table 1:** Specialties and size of clinics included in sample

	**Hospitals (beds)**				**Clinics (number of physicians)**	
	**< 300**	**400 to 700**	**400 to 700**	**> 700**	**≤ 10**	**> 10**
Cardiology			1			
Dermatology	1				2	1
Geriatrics			1			
Gynecology		1		1		
Internal medicine	1	1				
Ophthalmology	1	1		1		1
Orthopedics					2	2
ENT						1
Pediatrics				1		1
Plastic surgery		1	1		1	
Radiology						1
Surgery	1		1			
Total	4	4	4	3	5	7

In the interviews, respondents were first informed about the aim and method of the study. In order to avoid socially desirable answers as much as possible, they were explicitly asked to reflect on their actual experiences in their present situation in the hospital or specialty clinic. The interviews were audiotaped and transcribed, and then anonymized for reasons of confidentiality.

### Data analysis

The interviews were coded and analyzed with the help of Atlas.ti 6.2. The code list was based the constructs included in our research model (Figure 1). New elements that were mentioned as influencing intra-organizational dynamics, such as organizational size and personal characteristics, were also included in the code list.

In order to prevent bias, a second coder reviewed the recorded interviews as well. This resulted in a kappa of 0.73, showing relatively high inter-coder reliability. When differences arose, these were discussed; agreement was reached on all items. We translated the quotations included in this article from Dutch into English.

Next, we will compare our findings in hospitals with our findings in specialty clinics to determine whether entrepreneurship constitutes transformational or sedimented change, and we will present a refined model explaining physicians’ entrepreneurship.

## Results

First, we will establish the nature of intra-organizational dynamics in hospitals and in clinics. For this purpose, we will analyze the dynamics for each of the concepts in the research model: power dependence, interest dissatisfaction, value commitment, and entrepreneurship. Finally, we also provide some additional findings with respect to hospital entrepreneurship and elaborate on the type of change associated with the entrepreneurial process.

### Power dependence

We found that all physicians reported experiencing power dependencies and interest dissatisfaction with management or colleagues. The perceived high level of bureaucracy and lack of influence on hospital policy is frequently mentioned as causing interest dissatisfaction with management. With respect to this perceived level of involvement in decision-making, we found differences depending on the size of the hospital in relation to the management style. For example, physicians working in the smallest hospital included in our sample mention the culture of facilitating physicians. In the three other hospitals, the executive managers deal with matters differently, using formal and sometimes bureaucratic decision-making processes. In medium-sized hospital C, formal processes are perceived as dominating:

‘*I’ll suggest something and nothing gets done with it, you’re one month further and you think, oh I never heard anything more about that. That makes it even more of an uphill struggle.’* (*Manager, hospital C*)

In contrast, the management of the other medium-sized hospital B is more welcoming towards physicians’ initiatives as the executive manager takes a very open, pragmatic approach towards entrepreneurial initiatives by physicians, resulting in a relatively high number of initiatives executed in partnership with the hospital:

‘*As a rule, the medical specialists are more entrepreneurial than the managers. Managers think more in terms of limitations than physicians do. But if that’s your attitude (…) you’re literally not able to start something until you have a project assignment.*’ (*Executive, hospital B*)

Finally, the executive manager of the largest hospital (hospital D) included in our sample created a parallel informal process next to the standard bureaucratic process to allow for more direct input by physicians:

‘*Once every two years the entire Executive Board and directors and all the medical staff withdraw somewhere to brainstorm about a number of topics. That works much better than all that paperwork and it generates renewed dynamism.*’ (*Executive, hospital D*)

This parallel policy-making process helps physicians fulfil their interests. As a result, physicians’ interest dissatisfaction is relatively low. In addition, in the Netherlands most physicians work in physicians’ partnerships within a hospital instead on an employment basis. In fact, most of these physicians regard an employment relationship with the hospital as detrimental to their autonomy, causing a higher level of power dependence compared to an independent relationship:

‘*I’ve seen several employment relationships go bad in the hospital because (…) the directors first said yes, but then no, we mustn’t do this after all. And that leads to conflicts and in the end the director says, hang on a minute, I’m the boss here, remember?*’ (*Physician, hospital D*)

Summarizing, all physicians in hospitals experienced power dependence to some extent. Bureaucracy and a lack of perceived influence in the decision-making processes of management are highly influential factors. These factors in turn are dependent on hospital and partnership size and the managerial style of the executive manager. Hereby, more openness by management and greater facilitation of physicians are seen as ways of diminishing physicians’ perceived power dependence. Finally, an employment relationship between physicians and a hospital is regarded as causing greater power dependence.

For specialty clinics, we found that physicians involved in the entrepreneurial process and having a position in the clinic’s governance perceive the facilitation provided by management as good. A general manager of a specialty clinic explains his facilitation of physicians as follows:

‘*We set up everything to make it as easy as possible for the physicians to do their job. Indeed, they’re the boss. In that sense they’re not being ordered around by a manager. So they have an awful lot of say in how they can optimize their work and arrangements.*’ (*Manager, clinic #3*)

However, in clinics, organizational size also appears to be positively related to power dependence as physicians working in larger clinics mention power dependence more frequently. In contrast, respondents from smaller clinics experience more direct collaboration by being more centrally positioned within the organization. A manager of the largest clinic reports the downside of a large span of control:

‘*People always complain a lot. And the complaints are mainly about how remote the directors are - not me, but the directors: people think they are too distant.*’ (*Manager, clinic #2*)

In addition to organizational size, the single focus in most clinics on one specialty improved the perceived influence on organizational decision-making. As a result, internal competition with other specialties is limited or absent. This focused character contributes to a lower perceived power dependence.

Moreover, perceived power dependence is reduced by the financial incentives provided by specialty clinics. These incentives are related to the overall performance, thereby fostering a shared interest among physicians and managers alike. As a physician explains:

‘*We set up the clinic to give everyone a share in the profits. That makes you slightly more motivated to make sure things work out in your own outfit because it’s in your own interests.*’ (*Physician, clinic #4*)

With regard to physicians who were not involved in the entrepreneurial process of starting up the clinic and who are not included in the clinic’s governance, our results indicate that they still perceive high power dependence on management. In contrast, physicians who were involved in the startup phase and who are part of the governance structure of the specialty clinic perceive themselves to be more influential as well as seeing functional interdependence with - rather than dependence on - management. This mutual interdependence is perceived as a positive and vital characteristic of their organization, as a physician involved in the entrepreneurial process notes:

Summarizing, specific organizational characteristics of specialty clinics stimulate the development of shared interests and collaboration. For example, small specialty clinics without much bureaucracy can be more flexible with regard to physicians’ interests due to a smaller span of control for the clinics’ management and a decision-making process that involves a single specialty. In addition, many specialty clinics offer shared financial incentives, thereby aligning the interests of physicians and managers. Finally, physicians who have been involved in the founding of the clinic and who have a position in the clinic’s governance experience low levels of power dependence. Both physicians and managers are aware of their interdependence, which produces a context where physicians are facilitated in practicing their medical profession as autonomously as possible.

### Interest dissatisfaction

We found mixed results for the causes and extent of interest dissatisfaction among physicians. The causes of interest dissatisfaction with hospital management are often related to the perceived degree of red tape in decision-making. Some managers frankly acknowledge this:

‘*I often still need to work on creating the basic organization, so you don’t even get round to entrepreneurial activities, you’re too busy managing.*’ (*Manager, hospital C*)

A physician in hospital C explains his experiences with management:

‘*I’ve suggested a couple of really interesting opportunities to the hospital. But you never hear anything more about them. (…) I can’t understand from a rational viewpoint why a hospital doesn’t seize those opportunities’* (*Physician, hospital C*)

Good facilitation by management, on the other hand, was found to limit interest dissatisfaction among physicians, as illustrated by one physician:

*‘I would say the answer to the question why I don’t undertake entrepreneurial activities outside the hospital is that we basically have a good outpatient clinic and I’m satisfied with that.*’ (*Physician, hospital A*)

Besides hospital management, interest dissatisfaction may also be caused by colleagues, depending on the relative power dependence experienced with regard to either hospital management or other physicians. As most hospital-based physicians work in a partnership with other physicians, these partners are very important. Difficulties in this relationship can easily lead to conflicts and may result in entrepreneurship by the departing physician, as explained by a physician who was in the process of starting his own clinic after having left the hospital:

‘*If there comes a point that it’s blindingly obvious they want to get rid of you but don’t actually say so, (…) then I’m not inclined either to say well, I’ll just stay put. I can’t function if that kind of thing is going on.*’ (*Physician hospital x*)

Summarizing, we found mixed results on the causes and origins of interest dissatisfaction. Interest dissatisfaction of hospital-based physicians was related to the hospital’s bureaucracy and the resulting lack of opportunity exploration. On the other hand, good facilitation decreases interest dissatisfaction. Additionally, we also found an example of one physician with a high degree of perceived power dependence with regard to his colleagues. Due to the problematic relationship, this resulted in interest dissatisfaction with his fellow group members rather than with management.

In clinics, on the other hand, ten out of twelve physicians said they were satisfied with the facilitation provided by their management, as they were able to satisfy their interests and practice their medical profession while safeguarding their medical autonomy. Especially, physicians who were involved in the founding and governance of their specialty clinic reported being able to align their clinic with their own interests. A general manager describes how physicians’ interests are facilitated:

‘*The physician-entrepreneurs want short lines of communication, a focus on patients, a personal business, not too big, fast, no waiting, not being constantly shunted from pillar to post, high-quality service (…). And the facilities for that are what we’ve essentially organized.*’ (*Manager, clinic #4*)

The interests of physicians and managers in clinics are found to be highly aligned, thereby preventing interest dissatisfaction and competing interests. Instead, shared interests focus on achieving high patient satisfaction, and the best possible care is realized through shared ownership or financial incentives to optimize the clinic’s overall performance. A general manager described the importance of having shared interests:

‘*In fact, you need to make sure the interests of the doctors and the clinic are aligned. If the interests aren’t aligned, they will inevitably end up in a conflict.*’ (*Manager, clinic #3*)

It is noticeable, however, that as clinics grow, physicians and managers report experiencing a similar divergence of interests to managers and physicians working in hospitals. One physician who worked on an employment basis for a specialty clinic explains his difficult relationship with a manager:

‘*There comes a point where the management tells you, the doctor, how you should be doing your (…) and then you gradually get into disagreements with management.*’ (*Physician, clinic #7*)

Summarizing, physicians who participate in the founding and governance of the clinic are able to practice their medical profession while satisfying most of their interests; they experience low levels of interest dissatisfaction. Physicians’ interests are aligned with clinics’ overall performance through shared ownership or additional financial incentives. As clinics grow, however, the interests of managers and physicians start to diverge, thereby creating similar tensions as in hospitals.

### Logics and value commitments

Physicians embedded in the business-like logic or hybrid logic (containing elements from both the medical and business logic) experienced interest dissatisfaction with fellow physicians exhibiting *prima donna* behaviour rather than with hospital management. This results in a transformative oriented competitive value commitment, aiming to change the traditionally dominant logic of the own group. A physician embedded in a hybrid logic illustrates this:

‘*I try to communicate in their (managers’) language and not retreat into my medical ivory tower (…). At the same time there are an awful lot of doctors with really unreasonable demands. If someone like that cries out, “Good gracious, I’m the doctor here, what on earth are you thinking of?”.* If *you do that just once, you’ve lost your commitment for the next five years.*’ (*Physician, hospital A*)

For physicians embedded in the medical logic however, interest dissatisfaction is related to their perceived high power dependence on hospital management. Their decision to engage in entrepreneurship is based on a defensive oriented competitive value commitment aimed at upholding values derived from medical professionalism. As one physician states:

‘*I didn’t want to have to say “no” to patients who needed a simple operation just because that operation didn’t fit in with the management’s ideas.’* (*Physician, hospital B*)

Summarizing for hospitals, being embedded in the medical logic is associated with interest dissatisfaction with hospital management, while being embedded in a hybrid or business logic is associated with interest dissatisfaction with fellow physicians.

In clinics, we found a tendency for the development and maintenance of a shared hybrid logic held by both physicians and managers as part of the entrepreneurial process. The basic premise of this hybrid logic entails both groups adopting elements of the logic traditionally held by the other group. As a physician describes it:

‘*Just as managers need to learn to think a bit like a doctor - they need to be able to empathize with how doctors think - in the same way, doctors need to be able to empathize with how managers think.*’ (*Physician, clinic #6*)

One physician, who held a medical logic, did not participate in the entrepreneurial process of starting up the clinic and was not involved in the clinic’s governance collided with the hybrid logic and eventually left the specialty clinic;

‘*I had an office manager there. (…) I said: you need to facilitate me. And at a certain point, it was something really simple, I said that I wanted to arrange my consultation hours like this. “Yes, but we don’t agree.” Sorry, but if so then we have a misunderstanding.*’ (*Physician-manager, clinic #7*)

In addition, the relatively small size of specialty clinics helps in building and maintaining a shared hybrid logic as communication can be quick and direct, as explained by a manager:

‘*We regularly discuss the set of instruments doctors need, agreeing on communication with patients and how we should arrange procedures and protocols.*’ (*Manager, clinic #5*)

Finally, as the clinic expands, the business logic of management becomes more dominant at the expense of the hybrid logic, potentially giving rise to a defensively oriented competitive value commitment. A physician embedded in the hybrid logic described his feelings about the growth of his clinic:

‘*But you see that specialty clinics are getting bigger and bigger (…) and if they get big enough, you automatically get the same organizational problems in the specialty clinics as in the hospitals.*’ (*Physician, clinic #4*)

Likewise, the executive manager of hospital B comments on the dangers of the entrepreneurial entity becoming more bureaucratic:

‘*The management side has a tendency to make things more bureaucratic, they add a layer of bureaucracy to the opportunity and if you do that thoroughly then you inevitably find the opportunity has gone again.*’ (*Executive Manager, hospital B*)

Summarizing, at most of the specialty clinics included in our sample, physicians and managers are embedded in a shared hybrid logic. As physicians perceive their interests to be properly facilitated, their value commitment is directed towards maintaining this *status quo*. One physician who did not participate in the entrepreneurial process and the governance of the clinic collided with the dominant hybrid logic, thereby developing a defensive oriented competitive value commitment. Finally, we found that as clinics grow, so does the dominance of the business logic.

### Entrepreneurship

Based on our research model, we found two ways in which intra-organizational dynamics in hospitals contribute to physicians’ entrepreneurship. First, physicians embedded in the medical logic experience power dependencies and interest dissatisfaction with regard to management, which induces them to leave the hospital. Second, physicians embedded in the business logic experience power dependencies and interest dissatisfaction with regard to other physicians. However, we did not find examples of physicians embedded in the business-like health care logic whose decision to turn into an entrepreneur was induced solely by intra-organizational dynamics. Instead, we found other factors influencing physicians’ entrepreneurship. Coming from an entrepreneurial family facilitates the transition, as explained by a plastic surgeon embedded in the business logic:

‘*Ever since I was a child I’ve been brought up in an entrepreneurial environment (…). And then I started thinking: well, we could do that here in this hospital as well; set up our own business and deliver care privately ourselves.*’ (*Physician, hospital B*)

Another deciding factor for entrepreneurship is having an entrepreneurial nature, as described by a physician embedded in the business logic:

‘*I turned from being a doctor into an entrepreneur because it’s in my blood (…) it was just a question of acting according to your nature.*’ (*Physician, clinic #9*)

And as perceived by an executive hospital manager:

‘*Dissatisfaction and criticisms of the senior management, the Executive Board, confidence issues and all sorts of things - that can be one of the reasons for starting entrepreneurial activities. But you need to be a genuine entrepreneur to actually be able to take that step.*’ (*Manager, hospital B*)

For physicians already working in entrepreneurial specialty clinics, we only encountered one physician who therefore experienced power dependencies and interest dissatisfaction with regard to management, inducing him to leave the specialty clinic to start his own business. This physician was embedded in the medical logic and did not participate in the entrepreneurial process or in the clinic’s governance. We neither found evidence for high levels of power dependencies and interest dissatisfaction with the clinic’s management among physicians who did partake in the entrepreneurial process and governance of the clinic, nor did we encounter similar phenomena with respect to other physicians. Still, growing bureaucratization is mentioned as a potential danger to this stable situation as this may induce growing dependence on the clinic’s management, therefore resulting in interest dissatisfaction.

### Additional findings on hospitals

An important factor influencing the form of physicians’ entrepreneurship in hospitals is management’s basic attitude towards physicians’ entrepreneurship and management’s willingness to facilitate entrepreneurial initiatives (see Table 2). The management of three of the hospitals included in our sample only allows physicians’ entrepreneurship under certain conditions. Examples of these conditions include the requirement that the new clinic be located at a minimum distance from the hospital, and shared ownership, often with a majority stake for the hospital. For physicians who are dissatisfied with the facilitation provided by hospital management, this stance provides additional ‘evidence’ reinforcing this dissatisfaction.

**Table 2:** Level of competition, managerial attitude and actual initiatives

**Type**	**Level of competition**	**Managerial attitude towards entrepreneurship**	**Entrepreneurship (intra/extra)**
Small hospital	Low	Conditional	1/0
Medium hospital	High	Positive (vehicle)	5/1
Medium hospital	High	Conditional	1/2
Large Hospital	Low	Conditional	1/1

The management of the fourth hospital included in our sample was prompted by a perceived threat from a new large-scale competitor in the neighborhood to create an entrepreneurial vehicle as part of the hospital’s holding company structure. This vehicle was able to facilitate physicians attracted by the prospect of starting an entrepreneurial entity. As part of the arrangement, the hospital would hold a majority stake in every newly created entity.

‘*If you see what the specialists do with this, it’s not that much at hospital B. But what it has done is to send a message to the staff that (…) we also give them the freedom to be entrepreneurs. What you also see is that some specialists are so entrepreneurial that they say: I’m just going to start up my own businesses.*’ (*Manager, hospital B*)

Indeed, the facilitation provided for entrepreneurial initiatives has been no panacea preventing physicians from leaving this particular hospital, as one physician explains:

‘*I wasn’t interested in fitting in with a hospital hierarchy where ophthalmologists are somewhere near the bottom. It was time for something different, so I changed direction completely.*’ (*Physician, hospital B*)

In sum, we found most management teams *de facto* discourage physicians’ entrepreneurship by setting conditions. For physicians who are already dissatisfied, this increases their dissatisfaction with hospital management. In contrast, creating an entrepreneurial vehicle as part of the hospital may facilitate physicians in carrying out their initiatives. This, however, is no panacea preventing entrepreneurial physicians from leaving the hospital.

### Resulting type of change in clinics

Next, to assess whether the change to entrepreneurship was transformational or sedimented, we analyzed intra-organizational dynamics in specialty clinics. We found that physicians and managers who jointly started a specialty clinic developed a hybrid logic during the startup phase. This hybrid logic subsequently supports their collaboration as it becomes the dominant logic once the clinic is operational. Interestingly, physicians who are not involved in either the startup or governance of the specialty clinic still report high perceived power dependence on, and interest dissatisfaction with, clinic management. Instead of adopting the dominant hybrid logic, these physicians remain embedded in their traditional medical logic, thereby developing a defensive oriented competitive value commitment towards the hybrid logic. This finding suggests that involvement in the startup phase and governance itself comprises a sedimentation process, allowing physicians traditionally embedded in the logic of medical professionalism to adopt elements of the business-like health care logic, thereby developing a hybrid logic shared with management. The nature of intra-organizational dynamics in both hospitals and specialty clinics is summarized in Table 3.

**Table 3:** Characteristics of intra-organizational dynamics in hospitals; based on interviews of 15 physicians and 8 managers

							**Entrepreneurship**	
**Hospital**	**Beds**	**Level of competition**	**Perceived involvement in decision-making**	**Power dependence on management**	**Interest dissatisfaction with management**	**Management’s attitude towards entrepreneurship**	**Intrapreneurial**	**Extrapreneurial**
A	< 300	Low	Medium	Medium	Low	Negative	1	0
B	400 to 700	High	Medium	Medium	Low	Positive (Vehicle)	5	1
C	400 to 700	High	Low	High	High	Conditional	1	2
D	> 700	Low	Medium	Medium	Medium	Negative	1	1

We found two indications for the change being sedimented rather than transformational. As part of the medical logic, physicians are supposed to be centrally positioned in the organization allowing them to be both influential as well as autonomous [17]. Being involved in the startup process and governance of the specialty clinic and subsequently having direct influence on clinics’ policies not only diminishes the likelihood of interest dissatisfaction developing but also fits well with the initial medical logic in which most entrepreneurial physicians were initially embedded. This is supported by a growing body of literature pointing to the added value of involving physicians in governance [19,34-39]. In sum, physicians’ satisfaction in specialty clinics is not a result of a newly created logic resulting from transformational change, but rather the outcome of a good fit with the medical logic in which the physician was formerly embedded and which prevailed during the entrepreneurial process.

As noted by Pinnington and Morris [29], whereas transformational change is permanent, sedimented change is temporary and can be reversed. We found this to be the case with respect to the hybrid logic of small, focused specialty clinics. As illustrated by Mintzberg [40] and more recently by Marquis and Lounsbury [41], small organizations tend to turn into bureaucracies as they grow, thereby providing the conditions for an increasingly dominant business logic. As organizational growth requires more coordination, the business logic increasingly becomes dominant, thereby resembling the very organizations entrepreneurial physicians left in the first place. As a result, organizational members with a strong need for autonomy may leave the specialty clinic [42].

Finally, in specialty clinics physicians’ and managers’ interests are aligned by providing incentives. Shared interests have been shown to positively affect organizational performance and collaboration [20,37,38,43-46]. In fact, these incentives constitute additional cement to the sediment in which entrepreneurial values are ‘glued’ onto the central medical logic of physicians. This glue may also improve the fit between managers and physicians in other settings, like hospitals.

## Discussion

We explored the nature of intra-organizational dynamics between physicians and managers in both hospitals and specialty clinics. In addition, we assessed whether the change whereby a physician turns into an entrepreneur is largely transformational or sedimented.

A perceived high level of bureaucracy and the associated lack of opportunities for physicians to have a say on hospital policy were found to cause both perceived power dependence and interest dissatisfaction. In addition to the focus by Greenwood and Hinings (1996) on intra-organizational dynamics between functionally different groups leading to radical change, we also found evidence that intra-group dynamics may result in radical change, thereby inducing physicians to turn into entrepreneurs.

The initial embeddedness in a certain logic seems to determine physicians’ value commitment and related primary focus of perceived power dependence and interest dissatisfaction. Physicians embedded in the traditional logic of medical professionalism perceive high levels of bureaucracy and related power dependence on, and interest dissatisfaction with hospital management, resulting in a defensively oriented competitive value commitment. Moreover, intra-organizational dynamics constitute the main incentive for change, which takes the shape of entrepreneurship. Simultaneously, management’s attitudes to these types of initiatives by physicians influence how they will be implemented. If a hospital’s management explicitly discourages entrepreneurial initiatives, physicians face no alternative than to execute their entrepreneurial initiative outside the hospital, while if management is more facilitating, physicians will prefer to collaborate with the hospital organization in the form of intrapreneurship.

As a result, we conclude that our research model holds for physicians embedded in the logic of medical professionalism thereby developing a defensively oriented competitive value commitment. This confirms earlier findings of Marquis and Lounsbury [41], who found entrepreneurship to be stimulated by conflicting logics.

Yet, the physicians embedded in the dominant logic of business-like health care primarily reported interest dissatisfaction because of *prima donna* behaviour of fellow physicians embedded in the logic of medical professionalism unwilling to adapt to hospital policies. Thereby, a transformative oriented competitive value commitment is developed towards the traditionally dominant logic of physicians.

Although we found that intra-organizational dynamics encouraged entrepreneurship to take place, personal factors, in particular in the form of an entrepreneurial nature or nurture, were mentioned as incentive necessary conditions for the entrepreneurship of these physicians.

Finally, our finding that entrepreneurship represents sedimented rather than transformational change provides striking evidence for hybridized professionalism. As part of this concept, professionals are linked to other groups holding different logics [47] calling for new boundary spanning knowledge and skills. Until now however, hybridization was defined as professionals learning new behaviour to fit new and hybrid organizational forms [48]. Our research however indicates that in the process of resisting hybridization, new entrepreneurial entities can be found aimed to prevent hybridization by instead upholding values derived from the logic of medical professionalism. Surprisingly however, we found evidence that the entrepreneurial process aimed to prevent hybridization, instead entails a hybridization process by which elements of the ‘alien’ logic are adopted (Table 4).

**Table 4:** Intra-organizational dynamics in specialty clinics as compared with hospitals

	**Hospital**		**Specialty clinic**	
	**Physicians (n = 15)**		**Physicians (n = 12)**	
Logic	Medical (6)	Hybrid (1)	Medical (1)	Hybrid (6)
		Business (8)		Business (5)
Participated in startup			No	Yes
In governance			No	Yes
Perceived power dependencies^a^	High	Medium	High	Low
Prime subject of power dependencies	Management	Fellow physicians/Management	Management	-
Perceived interest dissatisfaction^a^	High	Medium	High	Low
Perceived value commitment	Competitive; defensive towards business	Competitive; transformative towards medical	Competitive; defensive towards business	*Status quo*

^a^‘Low’ when no example regarding perceived interest dissatisfaction or power dependencies is given, ‘medium’ when one or two examples are given, and ‘high’ when more than two examples are given.

### Study limitations and suggestions for further research

Although this was a qualitative study exploring aspects of intra-institutional dynamics, the responding selection of physicians and managers may have biased our findings. Given our relatively limited sample, a large-scale quantitative follow-up study is needed to confirm our qualitative findings. It remains unclear how the entrepreneurial process actually induces physicians to adopt elements from the business logic and how management is induced to adopt elements from the medical logic, thereby creating a hybrid logic. A follow-up longitudinal study should focus on this process of sedimentation and shed more clarity on the mechanisms involved. Finally, future research may extend our knowledge on the hybridization process itself including the mechanisms by which actors adopt new behaviours and skills.

## Conclusion

The main theoretical contribution of our paper lies in the conceptualization of our findings into a theory of entrepreneurial change in the health care sector. We drew from the neo-institutional theory developed by Greenwood and Hinings (1996), which assumed that organizations move to a coherent logic with a consistent set of structures and systems. Our analysis demonstrates that the hybrid entrepreneurial logic can be partially overlapping, consisting of different, and sometimes conflicting, layers from both the medical and business logic. As organizations grow, these layers may shift, thereby providing the impetus for a new cycle of change.

Rather than representing transformational change, our findings show that physicians’ entrepreneurship demonstrates the existence of sedimented change in which a hybrid logic held by both managers and physicians allows for a collaborative health framework in specialty clinics.

Our findings have implications for policy-makers both at the national level and the hospital level. At the national level, budget cuts resulting from the enduring economic downturn in Western countries have provided an impetus for new and additional health care reforms relying heavily on the logic of business-like health care. However in order to be effective, the deep involvement of physicians, decentralized decision-making and common ground with stakeholders embedded in the medical logic is needed, allowing for a hybrid logic to develop.

At the hospital level, policy-makers could learn from the mechanisms employed in specialty clinics. For example, by closely aligning organizational entities with medical specialties, management can be more focused and direct, simultaneously allowing for the greater influence and involvement of physicians. In addition, we found shared incentives for both management and physicians, based on both quality indicators and financial indicators of the entity’s performance, to be useful in providing additional cement to the sedimented hybrid logic.

## Competing interest

The authors declare that they have no competing interest.

## Authors’ contributions

WTK carried out the four case studies of the hospitals and five of the specialty clinics and participated in the analysis of the results and drafting of the manuscript. MR conducted seven of the case studies of specialty clinics and participated in both the analyses of the results and the drafting of the document. MLE helped to draft the final document. WHH participated in its design and coordination and helped to draft the manuscript. All authors read and approved the final manuscript.
